# Gold Nanoparticles Bioproduced in Cyanobacteria in the Initial Phase Opened an Avenue for the Discovery of Corresponding Cerium Nanoparticles

**DOI:** 10.3390/microorganisms12020330

**Published:** 2024-02-04

**Authors:** Melanie Fritz, Xiaochen Chen, Guifang Yang, Yuancai Lv, Minghua Liu, Stefan Wehner, Christian B. Fischer

**Affiliations:** 1 Department of Physics, University of Koblenz, 56070 Koblenz, Germany; 2 Fujian Provincial Engineering Research Center of Rural Waste Recycling Technology, College of Environment & Resources, Fuzhou University, Fuzhou 350116, China; 3 Materials Science, Energy and Nano-Engineering Department, Mohammed VI Polytechnic University, Ben Guerir 43150, Morocco

**Keywords:** *Anabaena* sp., biorecovery, biosynthesis, *Calothrix desertica*, digital image analysis, TEM

## Abstract

The production of isolated metallic nanoparticles with multifunctionalized properties, such as size and shape, is crucial for biomedical, photocatalytic, and energy storage or remediation applications. This study investigates the initial particle formations of gold nanoparticles (AuNPs) bioproduced in the cyanobacteria *Anabaena* sp. using high-resolution transmission electron microscopy images for digital image analysis. The developed method enabled the discovery of cerium nanoparticles (CeNPs), which were biosynthesized in the cyanobacteria *Calothrix desertica*. The particle size distributions for AuNPs and CeNPs were analyzed. After 10 h, the average equivalent circular diameter for AuNPs was 4.8 nm, while for CeNPs, it was approximately 5.2 nm after 25 h. The initial shape of AuNPs was sub-round to round, while the shape of CeNPs was more roundish due to their amorphous structure and formation restricted to heterocysts. The local PSDs indicate that the maturation of AuNPs begins in the middle of vegetative cells and near the cell membrane, compared to the other regions of the cell.

## 1. Introduction

The production of pure, stable, and isolated metallic nanomaterials with precise control over size, shape, and functionality is of paramount importance in many fields such as biomedical, photocatalytic, and energy storage or remediation applications [1,2,3]. Therefore, significant efforts are being made to identify microorganisms suitable as “nanobiofactories” for the green production of biogenic metallic nanoparticles (NPs) [4,5,6]. Biogenically synthesized nanoparticles (NPs) such as gold, silver, copper, and zinc oxides exhibit excellent enzyme inhibition, high biocompatibility, and antimicrobial and antioxidant activities. They can also be easily multifunctionalized with pesticides, herbicides, and growth hormones [7]. Therefore, biogenic NPs are an effective tool that could be widely used in the agricultural sector after the comprehensive risk assessment of their ecotoxicity [8].

Biogenic NPs can be used to remediate anthropogenic environmental pollution, such as heavy metal contamination [9]. Environmental remediation, particularly nano-remediation, is receiving attention due to its potential to revolutionize pollutant removal [10]. The possible mechanisms for remediating organic or inorganic contaminations include adsorption by nanoparticles or oxidation reactions with them [11]. Harmful algae-contaminated waters and wastewater polluted with pesticides or dyes can be remediated agroecologically by using biogenic NPs without further burdening natural biota [12]. The production of non-toxic, surface-functionalized, and monodisperse NPs using microorganisms enables excellent therapeutic applications, such as contrast agents for magnetic resonance imaging scans, biomarkers, cell labeling, drug and gene delivery, wound healing, and the treatment of cancer or pathogenic infections [13]. The key advantage of biogenic NPs is their small size, which allows them to better reach the application site and act in a more targeted manner, reducing unwanted side effects. This eco-friendly biosynthesis offers significant opportunities for innovative applications in food processing, preservation, packaging [14], resource recovery [15], and wastewater treatment [16]. This is due to their remarkable properties at the nanoscale, including small particle size, large surface-to-volume ratio, and adjustable morphological properties [17].

Algae belonging to the classes *Cyanophyceae*, *Chlorophyceae*, *Phaeophyceae*, and *Rhodophyceae* are capable of the intracellular biosynthesis of metallic NPs. This method of NP production is rapid, easy to handle, inexpensive, and nontoxic compared to conventional processes [4,18]. Bio-based synthesis using plants, bacteria, yeasts, fungi, or microorganisms is considered a “green” alternative that does not generate any hazardous waste or byproducts [19]. The customized NPs of different sizes, well-defined morphologies, and shapes can be generated cost-effectively by controlling the biosynthesis parameters such as temperature, the pH value, the incubation period (the time of exposure of the cells to the metal salt solution), salt concentrations, and the specific growth conditions of the bioreactor [20,21]. In the past, bio-based synthesis was disadvantaged compared to the conventional NP production methods due to the difficulty in obtaining size-specific NPs with controlled morphologies. However, recent studies have shown that certain factors can be influenced to affect particle size and shape, demonstrating that control is possible in principle [22,23].

Characterizing NPs below 25 nm has always been analytically challenging. Optical, electrical, magnetic, and mass spectrometry techniques are used for NP detection, but they all have limitations in terms of sensitivity, specificity, and reliability. Moreover, due to their small size, such NPs can penetrate cells and organs and even cross natural barriers such as the blood–brain barrier. This can make it challenging to detect and measure them in biological systems as they are embedded in the biomatrix, which can cause additional interference effects [24].

In order to obtain the accurate particle size distributions (PSDs), it is necessary to develop optimized methods and standard procedures for the detection and characterization of NPs in bioorganisms such as cyanobacteria. The further refinement of the current method to characterize the particularly tiny gold nanoparticles (AuNPs) produced in the cyanobacteria *Anabaena* sp. in the initial phase paved the way for the detection of the more difficult-to-detect cerium nanoparticles (CeNPs), which were synthesized under similar conditions in the cyanobacteria C*alothrix desertica*. Initially, AuNPs could only be identified at longer incubation times of up to 25 h, with no initial development at 10 h, because of the insufficient resolution or contrast. The formation of CeNPs takes longer, which is further complicated by the fact that the CeNPs formed have no sharp edges compared to their background due to their amorphous structure. This makes it more difficult to detect CeNPs than the other rare earth element NPs used in our previous studies [23,25,26]. This paper is dedicated to the previously established tool for the detection of AuNPs in the range down to ~2 nm, and the easy-to-use *Anabaena* sp. that forms them within 10 h, and CeNPs down to ~3 nm in *Calothrix desertica* formed within 25 h, respectively. Only a few studies deal with the error analysis caused by the digital size and shape analysis, which we also critically reflect on. The aim of the study is to develop an easy-to-handle, fast, and fully automatic analytical tool to precisely quantify the sizes and shapes of the bioproduced particles in the nanoscale range.

## 2. Materials and Methods

### 2.1. Cultivation of Cyanobacteria

The biosynthesis experiments of AuNPs and CeNPs were performed according to the previously established methods [22,23,25]. Briefly, the stock cultures of the cyanobacteria *Anabaena* sp. (SAG strain 12.82, Culture Collection of Algae, Göttingen, Germany) and *Calothrix desertica* (SAG strain 35.79, Culture Collection of Algae, Göttingen, Germany) with the corresponding Bold’s basal medium salt solutions of HAuCl_4_ or Ce(NO_3_)_3_ 6H_2_O, each 1 × 10^−4^ mol/L (ABCR GmbH, Karlsruhe, Germany), were grown under the optimal natural conditions before biomass was extracted from the media using centrifugation (16,000× *g*, 14,000× *g* rpm, 15 min) at the respective times (10 h for Au; 25 h for Ce). The extracted biomass was then carefully washed with deionized water. The liquid portion can be analyzed using inductively coupled plasma mass spectrometry (ICP-MS), while the solid portion was prepared for examination with transmission electron microscopy (TEM) [22,26].

### 2.2. Transmission Electron Microscopy (TEM)

Nanoscale imaging was performed with a HT-7700 TEM 7700 (Hitachi, Tokyo, Japan) using the high magnification and high resolution (HR) imaging mode. The system was operated at 100 kV acceleration voltage. The preparation procedure followed the established protocols and has been described in detail in previous studies [22,23,25].

### 2.3. Digital Image Processing (DIP)

The particle size distribution (PSD) was determined using the free software ImageJ 1.53d (W. Rasband and contributors, National Institute of Health, Rockville, MD, USA) with Java version 1.8.0_112 (64-bit) on a Windows 10 Pro system. The software identified all pixels contributing to a recorded NP. A Fujitsu Siemens H19-1 monitor with a resolution of 1280 × 1024 and refresh rate of 60,020 Hz was used for the evaluation. The color format was RGB, with an 8-bit depth and a standard dynamic range color space. The analysis was carried out with an Intel(R) HD Graphics 620 card.

**Figure 1:**
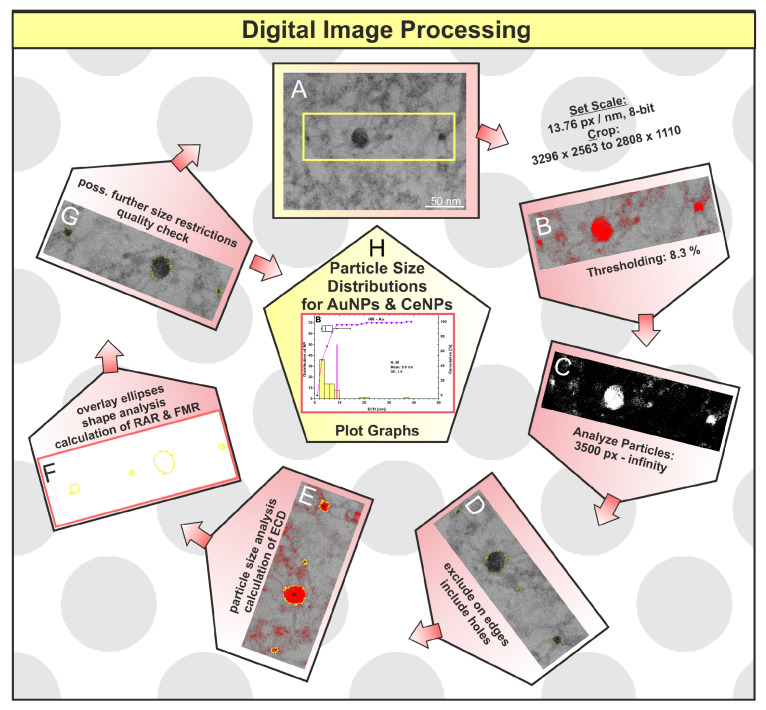
Individual steps (**A**–**H**) for analyzing the particle size and shape of gold and cerium nanoparticles using digital image processing (DIP).

Figure 1 briefly explains the steps of digital image processing (DIP). First, the TEM image was calibrated using the measuring bar. To better analyze the particles, the images were cropped, sharpened or smoothed, if necessary (Figure 1A), before the actual thresholding was applied (Figure 1B). Thresholding converts the 8-bit grayscale image to a 2-bit black and white image and evaluates contiguous pixels as particle areas (Figure 1C). Areas touching the edge are removed using the “exclude on edges” command, and missing pixels inside the particle are added using the “include holes” command (Figure 1D). The program marks registered particles with a yellow outline and outputs the corresponding numerical values of the red marked particle areas *A* and as the equivalent circular diameter (ECD, Equation (1), and Figure 1E). For a better understanding of this fact, the ECD is calculated assuming a perfectly round particle with the following formula:

ECD = (4 × *A*/π)^1/2^ (1)

Subsequently, ellipses are fitted (Figure 1F), on which the shape analysis is based, and the reciprocal aspect ratio (RAR, Equation (2)) and the Feret major axis ratio (FMR, Equation (3)) are calculated with the ellipse axes *a* (major) and *b* (minor) and the Feret diameter *D*_f_:

RAR = *b*/*a* (2)

FMR = *D*_f_/*a* (3)

Then, the original image is compared to the detected particles and checked for quality (Figure 1G). If necessary, manual adjustments such as further size restrictions can be made if particles have been misinterpreted. Lastly, the data are displayed with Origin Pro 8.5 OG SR1 software (Figure 1H), which has more features than those currently offered by the free ImageJ software. For statistical analysis, this software was used to calculate the mean and the standard error of the statistical mean. The detailed analysis of particle size and shape can be found in the authors’ previous study [25].

## 3. Results and Discussion

### 3.1. Exemplary TEM Images with Nanoparticles

**Figure 2:**
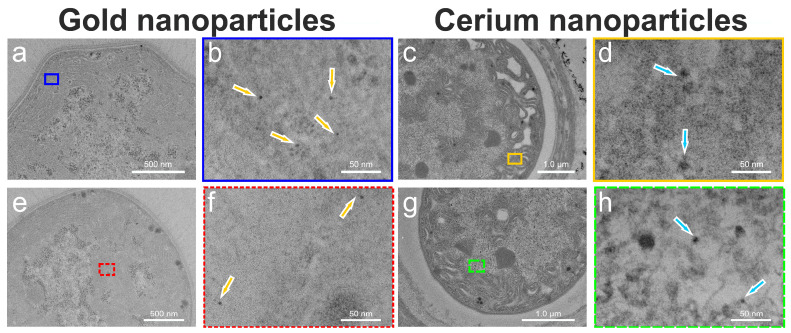
Exemplary TEM images of *Anabaena* sp. with gold nanoparticles (yellow arrows) in the vegetative cells (**a**,**b**,**e**,**f**) and cerium nanoparticles (light blue arrows) in the heterocysts of *Calothrix desertica* (**c**,**d**,**g**,**h**). For clarity, the respective areas of enlargement in (**b**) with blue outline are shown for magnification of (**a**), and in (**d**), yellow frame is for (**c**), and in (**f**), the red dots are for (**e**), and in (**h**), green dashes are for (**g**).

The cell sizes of cyanobacterial strains belonging to the genera *Anabaena* sp. and *Calothrix desertica* range from 3.5 µm^2^ to 19.3 µm^2^ (Supplementary Materials S.1 and Table S1). Ultrathin films (~60 nm) were used to section them. Heterocysts (HC) are specialized in N_2_ fixation and are surrounded by a thick cell wall, while photosynthesis occurs in the thin-walled vegetative cells (VCs) [27]. The TEM images in Figure 2 reveal two distinct VCs of *Anabaena* sp. (Figure 2a,e) that have produced biogenic AuNPs (Figure 2b,f indicated by yellow arrows) after a 10 h incubation. The inner AuNPs exhibit weaker contrast compared to the outer ones but are still detectable despite their small size of less than 10 nm in diameter. As the previous studies have suggested [22,26], dark condensations were also observed in the HCs of *Calothrix desertica* (Figure 2c,g) after a 25 h incubation. These irregular spots were identified as CeNPs in their initial stages using a high-resolution mode. Small CeNPs were detected inside the cells (Figure 2d,h, highlighted by light blue arrows).

**Figure 3:**
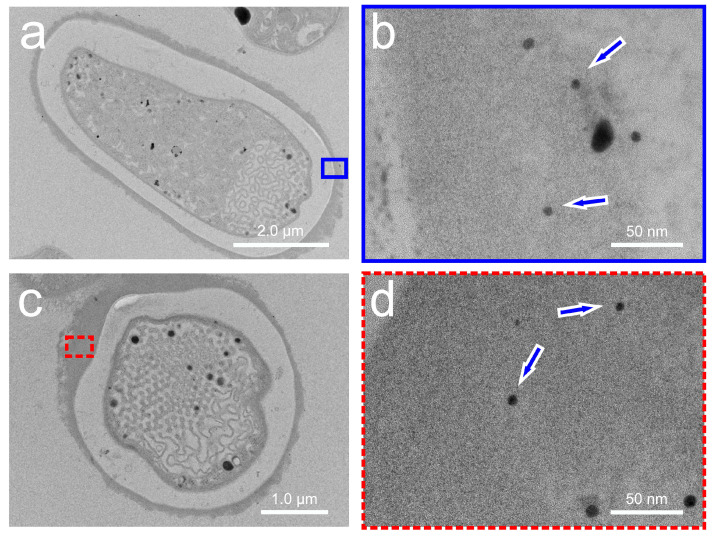
Gold nanoparticles are shown for heterocysts at low resolution (**a**,**c**) together with their indicated enlargements. The corresponding higher resolutions are shown in (**b**) for the blue-outlined area in (**a**), and in (**d**), for the red-dashed area in (**c**). Some gold nanoparticles are marked with blue arrows in (**b**,**d**), which are located exclusively outside the cell membrane of *Anabaena* sp.

AuNPs were not found inside the HCs (Figure 3), but at the periphery of the cell membrane of *Anabaena* sp., and this was already known from former studies [22]. The CeNPs resemble the amorphous structures of the nano-sized samarium (Sm) particles bioformed in *A. cylindrica* [25] or by europium (Eu) in *Anabaena* sp. at later growth stages [23].

### 3.2. Particle Size and Shape Analysis

#### 3.2.1. Biosynthesized AuNPs

**Figure 4:**
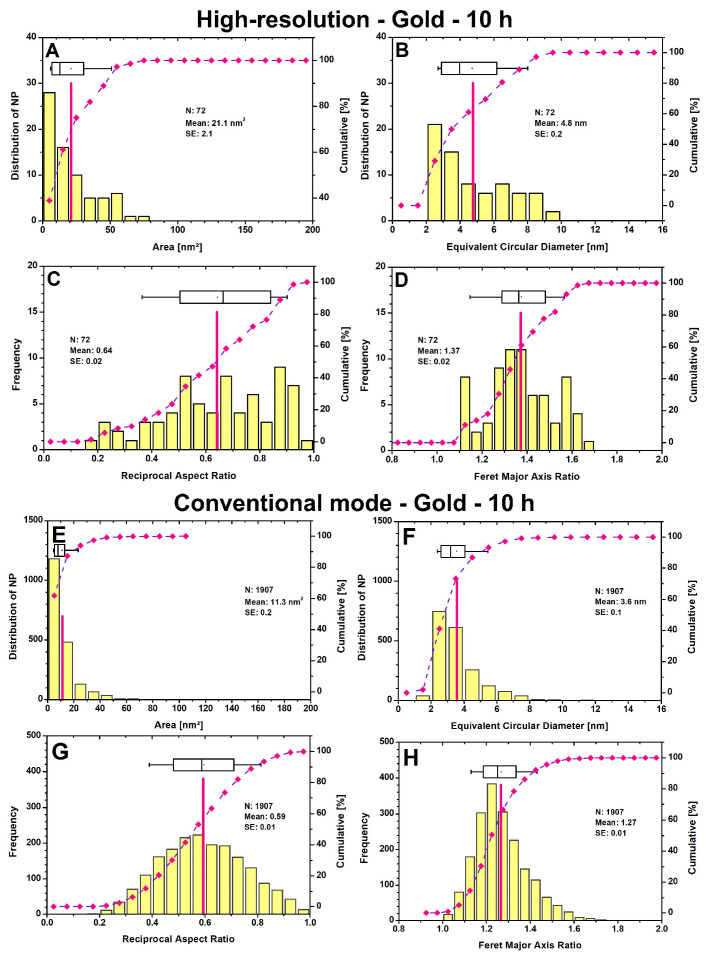
Particle size distribution and shape classification for gold nanoparticles in high-resolution mode (**A**–**D**) and in conventional mode (**E**–**H**) after 10 h are presented. The number (N) of nanoparticles, their mean (indicated by red lines), and their standard error (SE) are shown. The curve displays the cumulative value in percent, which refers to the right axis. Boxplots show the 25th and 75th percentiles, with whiskers at 10% and 90%.

The theoretical detection limit of high-resolution transmission microscopy (HR-TEM) is 0.05 nm, with modern TEMs operating at a theoretical detection limit of 0.20 nm in their conventional mode (CM). When particle size distributions (PSDs) are generated from lower resolution images using a conventional TEM, this inevitably results in higher deviations. The effect can be clearly seen in Figure 4. The 72 particles from the high-resolution (HR) mode, out of the 1907 AuNPs from CM, have an average area of 21.1 nm^2^ (Figure 4A), which is only 11.3 nm^2^ in the CM (Figure 4E). Converting to the equivalent circular diameter (ECD) and assuming that all particles are perfectly round, the diameter is minimized from 4.8 nm in HR to 3.6 nm in CM. A closer look at the PSD (Figure 4B) shows that in HR, half of the particles (50%) have an ECD between 2 and 4 nm, whereas in CM, this is 73% (Figure 4F). No particles smaller than 1.0 nm or larger than 10.0 nm were registered. The lower limit is restricted by the size range setting (Supplementary Material Tables S2 and S3), which depends on the image quality. It is accepted that smaller particles exist, but they cannot be clearly distinguished from the background noise, and therefore, their consideration is not very meaningful. The upper limit is usually infinite for HR and only needs to be readjusted for CM to exclude larger cell components as particles. The TEM images on which the PSDs in Figure 4 are based can be found in the Supplementary Material Figures S1 and S2. The shape classification indicates that the AuNPs have a mean reciprocal aspect ratio (RAR) of 0.64 for HR (Figure 4C) and a non-Gaussian distribution, compared to 0.59 for CM (Figure 4G). The interval assignment of the RAR values in Table 1 distinguishes the shape classes from “very angular” to “very round”. Therefore, the particle shapes are both in the round range, with a higher tendency towards very round for HR. The Feret major axis ratio (FMR), a shape parameter for PSD, has a mean value of 1.37 for HR (Figure 4D) and 1.27 for CM (Figure 4H), which highlights that some isolated particles have highly irregular structures. It is important to note that the HR images only capture a fraction of the particles present in the cell with high accuracy, while the CM can better reflect the total ratio of all particles, thereby sacrificing size accuracy.

Finally, it is worth considering a comparison with previous studies [23] that examined the PSDs for 24 and 51 h. The AuNPs had an average ECD of 8.4 nm for 24 h (160 particles) and 7.2 nm for 51 h (314 particles) in HR. The value obtained here for 10 h is significantly lower at 4.8 nm. Overall, smaller particles are initially formed after 10 h, whereby only 72 particles were registered in total, although more imaged material was evaluated. This indicates that probably fewer (detectable) particles were formed in a short time. After 10 h, the percentage of particles classified as “very round” reached almost 42%. After 24 h, over 88% of the particles are in the “very round” state.

**Figure 5:**
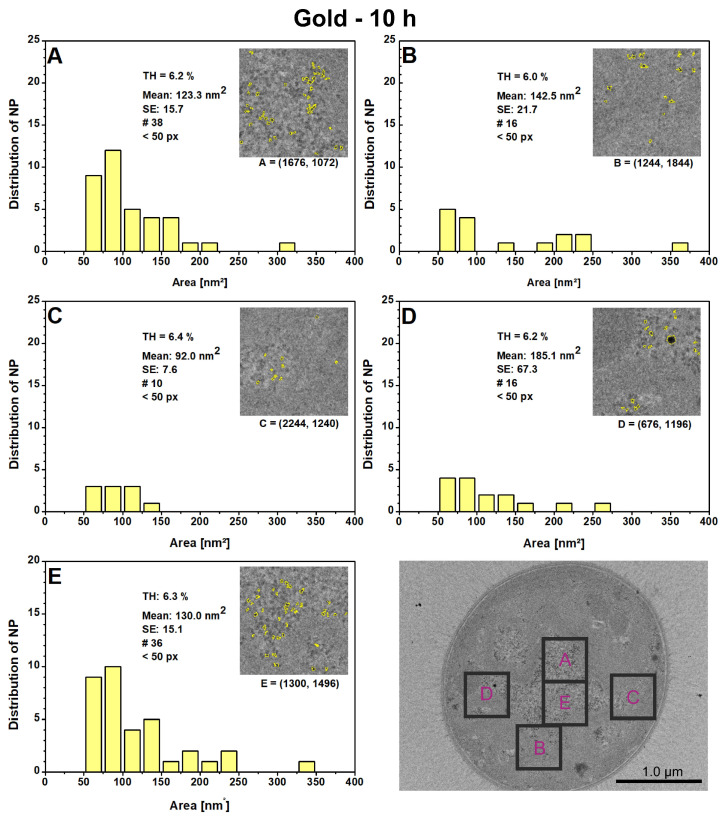
The local particle size distributions for gold nanoparticles were measured after a growth time of 10 h in five randomly selected 500 × 500 nm^2^ areas (**A**–**E**) within the presented vegetative cell. The plots display the thresholds (TH), mean with standard error (SE), the number of detected particles (#), the lower particle limit in pixels (px), and the local (x,y)-coordinates for each digital image processing.

To identify the local hotspots or agglomerations within cells, local PSDs were performed for five randomly selected 500 × 500 nm^2^ areas. Figure 5 presents these localized results for AuNPs after 10 h. The corresponding thresholds (THs) differ only slightly, as the selected locations have similar gray levels. To ensure consistency, a lower limit for the particle size (<50 pixels) was set. There was no upper limit, except for Figure 5D, where the largest hit (a black spot) was removed as an artifact. Areas A and E have a higher number of particles (A: 38; E: 36) than areas B and D with 16 each and C with 10. Most particles are typically found within the range of 50–100 nm^2^, with the majority falling within this range (A: 57%; B: 56%; C: 60%; D: 53%; E: 54%). Individual larger particles with a size over 300 nm^2^ can be found in areas, A, B, and E. These are probably closely spaced particles, as no particles were previously found in these size ranges. During the initial phase, particle growth appears to be more favorable in the central cell regions compared to the peripheral regions. This phenomenon is no longer present at 24 h and 51 h. The particles are now evenly distributed in the cell areas, in slightly varying amounts and more uniform in size, as explained in detail in reference [23].

#### 3.2.2. Biosynthesized CeNPs

**Figure 6:**
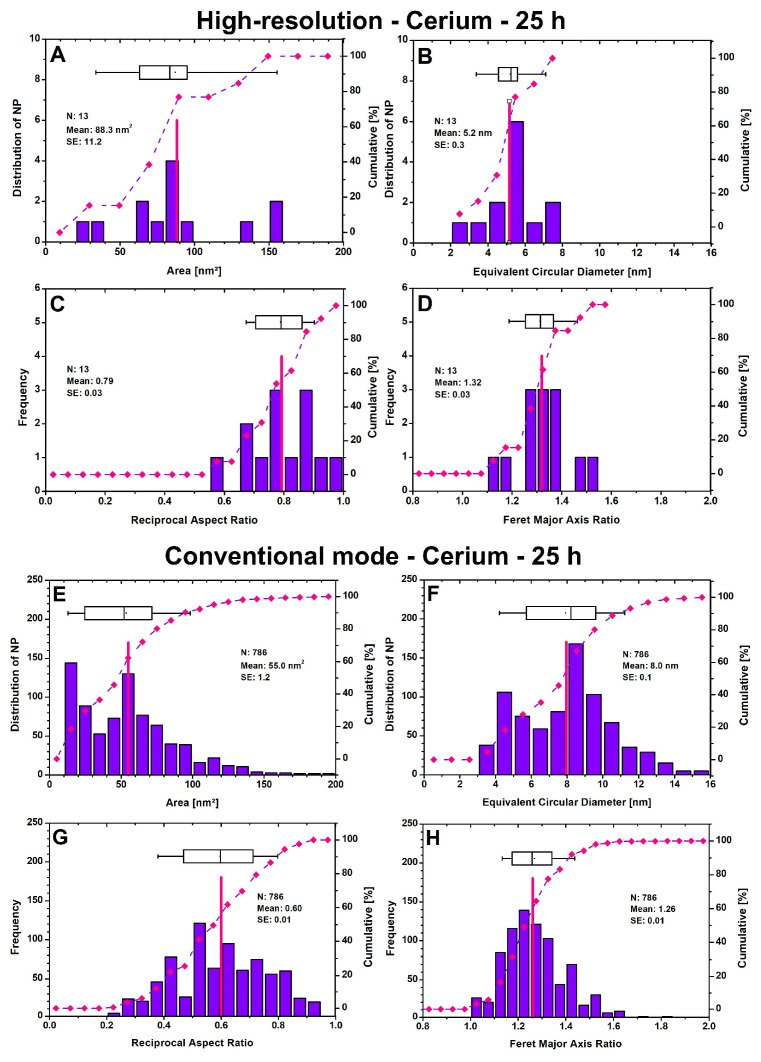
Particle size distributions and shape classification for cerium nanoparticles in high-resolution mode (**A**–**D**) and in conventional mode (**E**–**H**) after 25 h are presented. The number (N) of nanoparticles, their mean (indicated by red lines), and their standard error (SE) are shown. The curve displays the cumulative value in percent, which refers to the right axis. Boxplots show the 25th and 75th percentiles, with whiskers at 10% and 90%.

The TEM images in Figure 6 on which the PSDs are based and the parameters used for DIP can be found in the Supplementary Material (Figures S3 and S4 and Tables S4 and S5), respectively. Figure 6A shows that 13 CeNPs were finally detected, with sizes ranging from 26 to 160 nm^2^ and a mean of 88.3 nm^2^ at 25 h. The majority of the particles (~62%) fall within the size range of 60–100 nm^2^. The ECD was calculated assuming round particles, resulting in an average value of 5.2 nm (Figure 6B) in HR. The RAR value of 0.79 and the FMR value of 1.32 indicate that most of the particles are either ellipsoidal or round in shape (Figure 6C,D). The HR images only depict a small section of the particles, which were compared to the conventional mode, where 786 particles were finally registered. The comparison revealed that ca. 18% of the particles were smaller than 20 nm^2^, which was not the case with HR. A second predominant peak is observed in the 50–60 nm^2^ range, which accounts for almost 17% of the total particle number and lowers the mean value to 55.0 nm^2^. The average ECD is 8.0 nm. The most common ECD classes are in the range of 8–9 nm (21%), followed by 9–10 nm (13%) and 4–5 nm (14%). The range below 3 nm is considered negligible, as the inaccuracy increases due to the resolution limit. Additionally, an increase in the number of particles leads to less-rounded particles, as shown by the reduction in RAR from 0.79 (HR) to 0.60 (CM) and in FMR from 1.32 (HR) to 1.26 (CM).

**Figure 7:**
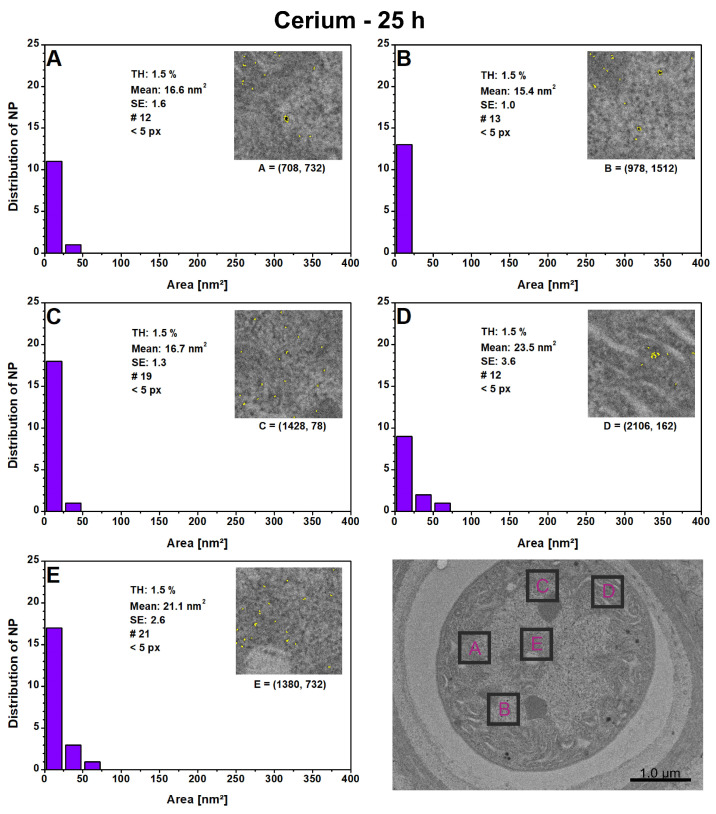
The local particle size distributions for cerium nanoparticles were measured after 25 h in five randomly selected 500 × 500 nm^2^ areas (**A**–**E**) within the presented heterocyst. The plots display the thresholds (THs), mean with standard error (SE), the number of detected particles (#), the lower particle limit in pixels (px), and the local (x,y)-coordinates for each digital image processing.

The comparison of the local PSDs in Figure 7 reveals that area E within the heterocyst has the highest number of particles, i.e., 21, and a slightly increased mean particle size of 21.1 nm^2^ (A:16.6 nm^2^; B. 15.4 nm^2^; C: 16.7 nm^2^). Area D shows a rather untypical accumulation of particles, which is why the highest mean value of 23.5 nm^2^ is observed here. The cell structure of the HC in area D also differs from the other areas and possesses a lamellar structure, which could originate from thylakoids. A comparison of the local PSDs for Europium NPs after 10 and 244 h and Samarium NPs after 25 h from previous studies [23,25] indicates that particle growth is favored in the cell interior and in the lamellar regions, with a lower occurrence of agglomeration. All intracellularly produced NPs in cyanobacteria are roundish and evenly distributed in the cells.

**Figure 8:**
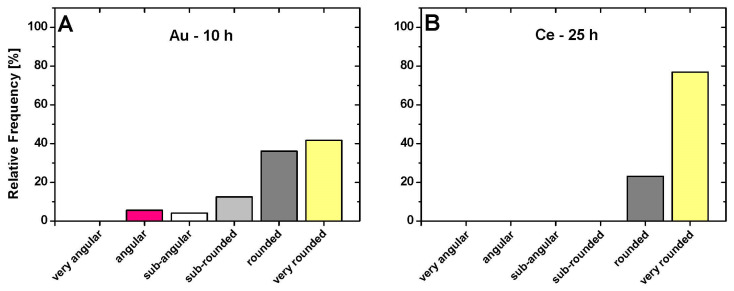
Classification into the six form categories for gold nanoparticles after 10 h (**A**) and for cerium nanoparticles after 25 h (**B**), with percentage rates.

Figure 8 shows that AuNPs in their early stages have lesser rounded shapes (angular: 5.6%, sub-angular: 4.2%, sub-rounded: 12.5%) than CeNPs, where all particles fall into the “rounded” or “very rounded” category. When compared to Europium NPs after 10 h, the CeNPs have a more uniform round shape. The results in Figure 8 only present a rough estimation of the data. To facilitate a better discussion, the evaluation technique should be further improved. It is uncertain whether the differences in the chemical standard properties such as the ionic radius, the preferred oxidation state, crystal structure, or the strain of cyanobacteria used as a biocontainer may affect particle size and shape.

#### 3.2.3. Quality Control

**Figure 9:**
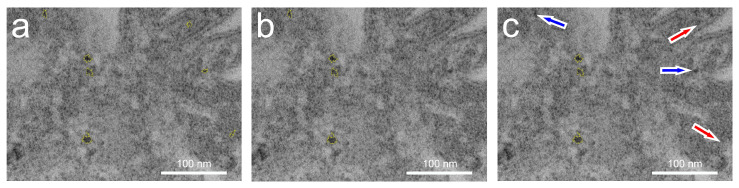
All cerium nanoparticle counted with a size restriction of 26–160 nm^2^ ((**a**), 7 hits), when the lower limit is shifted to 30 nm^2^ ((**b**), 4 hits) and 40 nm^2^ ((**c**), 3 hits) are framed in yellow. The red arrows indicate particles in (**c**) that were correctly disregarded, while the blue arrows indicate particles that were incorrectly disregarded in favor of higher hit reliability.

Through improvements in the digital image processing, the early stage AuNPs and the very small CeNPs below an ECD of 12 nm could be detected with various test runs for validation. For instance, Figure 9 illustrates the optimization measure. The TEM images not only show the particles but also cellular components, and some of which have the same gray values as the particles. During the test runs, the particles were narrowed down to a size range of 26–160 nm^2^ (Figure 9a). By adjusting the size restrictions, particles are removed or added. Due to the relatively small number of particles in the magnifications of the cell sections in the HR, scanning the entire cell to determine a reliable PSD is time-consuming. However, the HR images can provide important data for the accurate particle size, as the error rate is lower.

**Figure 10:**
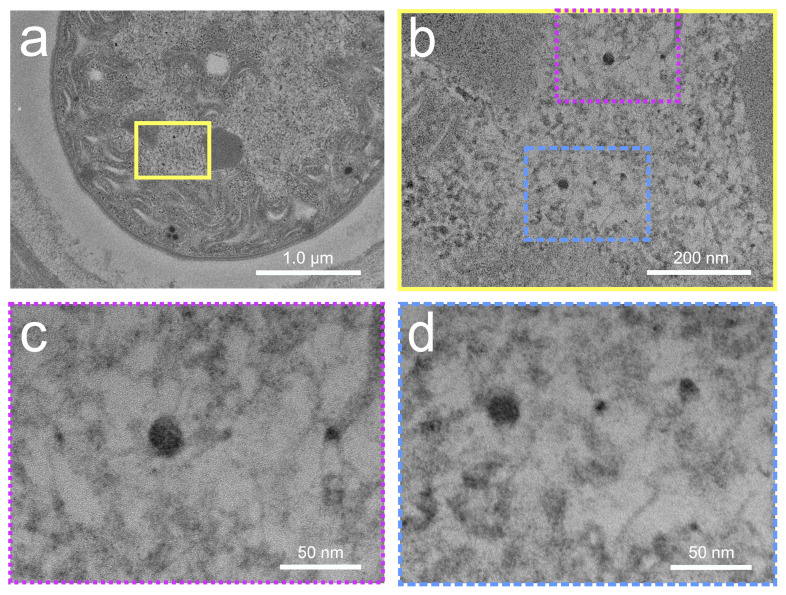
Two prominent cerium nanoparticle triplets in the heterocyst of *Calothrix desertica* (**a**) were magnified ((**b**) yellow framed) and further magnified ((**c**) purple dotted/(**d**) blue dashed) and compared to verify accuracy.

This prior knowledge serves as a basis for evaluating complete cells in the conventional imaging mode. This approach minimizes the susceptibility to errors and allows for the convenient acquisition of a larger amount of data with improved accuracy. Figure 9 demonstrates the value of the area restriction by correctly excluding some program hits that were not CeNPs. The restriction narrowed the hits down to seven (Figure 9a, yellow-framed particles), but one of them was still misinterpreted by the program. As a result, the lower limit had to be raised from 30 nm^2^ to 40 nm^2^. Unfortunately, this means that even correct CeNPs (Figure 9c, marked in blue) are occasionally disregarded. During testing of the interval of 30–40 nm^2^, the correct value was set to 36–160 nm^2^. The limit of one image is also applied to several other HR images to obtain an appropriate intersection for DIP of the TEM images in the conventional mode. The extent to which this restriction makes sense was double-checked. It is very likely that the same particles will be classified as different sizes on a different image with a different magnification (Mag.) factor. Therefore, additional tests were performed to verify the accuracy. In the final step, two prominent triplets of CeNPs from different images (Figure 10) were compared at three different magnifications (Mag. = 10,000, 50,000, and 150,000) before the PSD was analyzed with images showing half of the cells. The results of this comparison can be found in Table 2, where correctly analyzed values are assumed for Mag. 150,000 (see the next paragraph).

The data in Table 2 show that particle 1 with a significantly smaller value at 10,000 is obviously more prone to errors. However, particles 2 and 3 show good agreement with 150,000. The influence of the threshold value is demonstrated by the two right columns, with a threshold of 3.6% being too low and a threshold of 9.4% being acceptable. Particle 4 is 1.1 nm larger at Mag. 10,000 and 0.7 nm smaller at 100,000. In the case of particle 5, the ECD for 10,000 is also 0.7 nm larger compared to that at 100,000 and 150,000, and for particle 6, at Mag. 10,000, it is 1.0 nm larger than that at 150,000. The error susceptibility in the conventional mode at 10,000 is therefore approximately ±1.0 nm. Values below an ECD of 3.5 nm are typically disregarded during the automatic run. The program’s stability is the highest at a magnification of 150,000, which therefore serves as a reference.

The results of this test are somewhat subdued and show that a PSD performed on 10,000 images can only be regarded as a tendency and cannot provide more precise information on the exact particle sizes with an ECD smaller than 2.4 nm. This is especially true if the CeNPs have an amorphous structure, as is the case here. For the compact AuNPs, these issues are less critical. Generally, AuNPs are more easily detectable in biological microorganisms due to their strong surface plasmon resonance, which causes a noticeable color change when they aggregate or disperse. This color change can be observed through spectroscopic methods, provided that the AuNPs are large enough. Additionally, the nearly perfect round shape of AuNPs after a certain growth period allows for their detection without the risk of confusion with other cellular components. But, in the initial stage, the AuNPs have an ECD of less than 12 nm and are also not perfectly round in shape. In this case, their successful detection could only be achieved by improving the DIP method.

## 4. Summary

The current study shows that *Calothrix desertica* is a good candidate for CeNPs biosynthesis because it is known to be devoid of harmful neurotoxins, similar to *Anabaena* sp. for AuNPs, which also produces no harmful byproducts. It has been shown that particle growth is favored in the cell interior and in the lamellar areas, with less agglomeration. All cyanobacterial intracellularly produced NPs are round and evenly distributed in the cells with negligible particles below 3.0 nm due to resolution limitations. The detection of AuNPs with a diameter below 12.0 nm or very small CeNPs was achieved through improvements in the digital image processing. Test runs were carried out to reduce the particles to a size range of 26–160 nm^2^, allowing the better sorting and identification of particles. This method reduces susceptibility to errors and makes it possible to conveniently record a larger amount of data with improved accuracy. The area restriction was useful in excluding non-CeNP hits by reducing the lower limits. The accuracy of the DIP was confirmed by comparing two prominent particle triplets of CeNPs in the different images of three different magnifications. The method has a maximum error of approximately 0.4 nm at different magnifications (10 k, 100 k, and 150 k) of the same particle groups, which is noteworthy. This value considers fluctuating grayscale values by adjusting the threshold and illustrates the contrast between the conventional mode (CM) and the high-resolution (HR) mode. The study found that 72 AuNPs detected in HR had an average area of 21.1 nm^2^ out of 1907 particles in the CM with an average size of only 11.3 nm^2^. The determined diameter decreased from 4.8 nm in HR to 3.6 nm in CM. In HR, and half of the particles had an equivalent circular diameter between 2.0 and 4.0 nm, while in CM, it was over 73%. The shape classification revealed that the number of “rounded” and “very round” particles is up to 78%. Out of the 13 CeNPs detected in the size range of 26–160 nm^2^ with a mean of 88.3 nm^2^, the majority of particles (62%) were in the 60–100 nm range, with an average ECD value of 5.2 nm in the high-resolution (HR) mode. The PSDs and the shape classification of CeNPs were compared with HR and CM, resulting in only “rounded” to “very rounded” particles.

## 5. Conclusions

Future studies need to further investigate the time-dependent growth of size and shape of biogenic nanoparticles, taking into account the differences between the cyanobacterial strains used as biocontainers. More automated steps should be sought to obtain more reliable values when analyzing larger amounts of data. With regard to their application as a pollutant-removal material or as a biomedical material, studies are needed that assess their sustainability, efficiency, long-term behavior, and toxicity. Overall, cyanobacteria seem to be a reasonable alternative biocontainer to produce biogenic nanoparticles for these purposes.

## Figures and Tables

**Table 1:** The classification of reciprocal aspect ratio (RAR) values categorizing the six shape classes.

Class	Very Angular	Angular	Sub- Angular	Sub- Rounded	Rounded	Very Rounded
RAR value	0.12–0.17	0.17–0.25	0.25–0.35	0.35–0.49	0.49–0.70	0.70–1.00

**Table 2:** Two outstanding cerium nanoparticle triplets are identified (particles 1–3 and particles 4–6) and compared based on their equivalent circular diameter (ECD) at different TEM magnifications (150,000, 100,000, and 10,000). The correctly analyzed values are considered as a reference for the highest magnification of 150,000.

Magnification	150,000	100,000	10,000
Threshold [%]	8.0	9.4	3.6
Particle	ECD [nm]	ECD [nm]	ECD [nm]
1	3.0	3.1	2.4
2	11.8	11.0	11.6
3	5.3	4.8	5.4
4	10.2	9.5	11.3
5	3.3	3.3	4.0
6	5.0	4.6	6.0

## Data Availability

The raw and processed data required to reproduce these findings presented in this study are available on request from the authors.

## Supplementary Materials

The following supporting information can be downloaded at: https://www.mdpi.com/article/10.3390/microorganisms12020330/s1. S.1: Exemplary TEM images with nanoparticles; Table S1: Cell sizes of the vegetative cells (VCs), numbered VC-1 to VC-7, and heterocysts (HC), numbered HC-1 and HC-2, respectively; S.2: Biosynthesized gold nanoparticles (AuNPs); Table S2: The TEM image settings used to determine the size and shape of AuNPs in Figure 4 are presented below. The symbol “∞” represents infinity; Figure S1: The PSDs of the AuNPs and their shape classification in Figure 4 for the high-resolution mode are based on the TEM images shown here; Table S3: The settings of the respective TEM images showing AuNPs in the cells (shown for one magnification; here, 500 nm bar, VC); Figure S2: The PSDs of the AuNPs and their shape classification in Figure 4 for the conventional mode are based on the TEM images shown here; S.3: Biosynthesized Cerium nanoparticles (CeNPs); Table S4: The TEM image settings used to determine the sizes and shapes of CeNPs in Figure 6 are presented below. The symbol “∞” represents infinity; Figure S3: The PSDs of the CeNPs and their shape classification in Figure 6 for the high-resolution mode are based on the TEM images shown here; Table S5: The TEM image settings used to determine the sizes of CeNPs using the conventional mode; Figure S4: The PSDs of the CeNPs and their shape classification in Figure 6 for the conventional mode are based on the TEM images shown here.
