# Supplementary material for: Gold Nanoparticles Bioproduced in Cyanobacteria in the Initial Phase Opened an Avenue for the Discovery of Corresponding Cerium Nanoparticles
Source: Microorganisms. 2024 Feb 4;12(2):330. doi: 10.3390/microorganisms12020330 (PMC10892827; doi:10.3390/microorganisms12020330)
Supplement: Supplementary file 1 [file microorganisms-12-00330-s001.zip › microorganisms-2858835-supplementary.pdf]

Supplementary Material

Gold Nanoparticles Bioproduced in Cyanobacteria in the Initial Phase Opened an Avenue for the Discovery of Corresponding Cerium Nanoparticles

Melanie Fritz [1,*], Xiaochen Chen [2], Guifang Yang [2], Yuancai Lv [2], Minghua Liu [2], Stefan Wehner [1], Christian B. Fischer [1,3,*]

[1] Department of Physics, University of Koblenz, 56070 Koblenz, Germany
[2] Fujian Provincial Engineering Research Center of Rural Waste Recycling Technology, College of Environment & Resources, Fuzhou University, Fuzhou 350116, China
[3] Materials Science, Energy and Nano-engineering Department, Mohammed VI Polytechnic University, Ben Guerir 43150, Morocco
* Correspondence: fritz.melanie@web.de; chrbfischer@uni-koblenz.de
Tel: +49-261-287-2345, Fax: +49-261-287-100-2345

Content

S.1 Exemplary TEM images with nanoparticles page S2

Table S1 Cell sizes of the vegetative cells (VC), numbered VC-1 to VC-7, and heterocysts (HC), numbered HC-1 and HC-2, respectively.

S.2 Biosynthesized gold nanoparticles (AuNPs) page S3-S4

Table S2 The TEM image settings used to determine the size and shape of AuNPs in Figure 4 are presented below. The symbol "∞" represents infinity.

Figure S1 The PSDs of the AuNPs and their shape classification in Figure 4 for the high-resolution mode are based on the TEM images shown here.

Table S3 Settings of the respective TEM images showing AuNPs in the cells (showing for one magnification; here 500 nm bar, VC).

Figure S2 The PSDs of the AuNPs and their shape classification in Figure 4 for conventional mode are based on the TEM images shown here.

S.3 Biosynthesized Cerium nanoparticles (CeNPs) page S5-S6

Table S4 The TEM image settings used to determine the sizes and shape of CeNPs in Figure 6 are presented below. The symbol "∞" represents infinity.

Figure S3 The PSDs of the CeNPs and their shape classification in Figure 6 for the high-resolution mode are based on the TEM images shown here.

Table S5 The TEM image settings used to determine the sizes of CeNPs by conventional mode.

Figure S4 The PSDs of the CeNPs and their shape classification in Figure 6 for conventional mode are based on the TEM images shown here.

S.1 Exemplary TEM images with nanoparticles

Note that the size of the cells depends on the preparation of the ultrathin films, which are approximately 60 nm thick. The orientation of the cells may vary, and the cuts can be made horizontal (resulting in maximum size) or at any angle to the vertical (resulting in minimum size) relative to the cutting direction. AuNPs are produced in the vegetative cells (VC) of *Anabaena* sp. and CeNPs are produced in the heterocysts (HC) of *Calothrix desertica*.

Table S1 Cell sizes of the vegetative cells (VC), numbered VC-1 to VC-7, and heterocysts (HC), numbered HC-1 and HC-2, respectively.

**TEM image no.**	**Cell size [μm²]**
VC-1	4.8
VC-2	3.5
VC-3	3.6
VC-4	6.9
VC-5	5.9
VC-6	5.5
VC-7	6.5
HC-1	19.3
HC-2	9.0

S.2 Biosynthesized Gold nanoparticles (AuNPs)

Table S2 The TEM image settings used to determine the size and shape of AuNPs in Figure 4 are presented below. The symbol "∞" represents infinity.

TEM image no.	Set scale factor [px/nm]	Particle counts	Size range [px]	Threshold [%]
1	13.76	5	1200-5000	5.64
2	13.74	5	1500-5000	5.36
3	13.74	2	5500-5700	21.04
4	13.74	1	300-∞	5.22
5	13.76	13	1000-1500	10.19
6	13.74	11	1000-1500	14.11
7	13.78	2	2200-5000	5.88
8	13.74	7	2000-3000	8.54
9	13.74	1	100-∞	3.21
10	13.74	14	3500-10000	14.50
11	13.76	5	6000-∞	10.08
12	13.74	5	1500-∞	13.31
13	13.76	1	50-∞	2.16

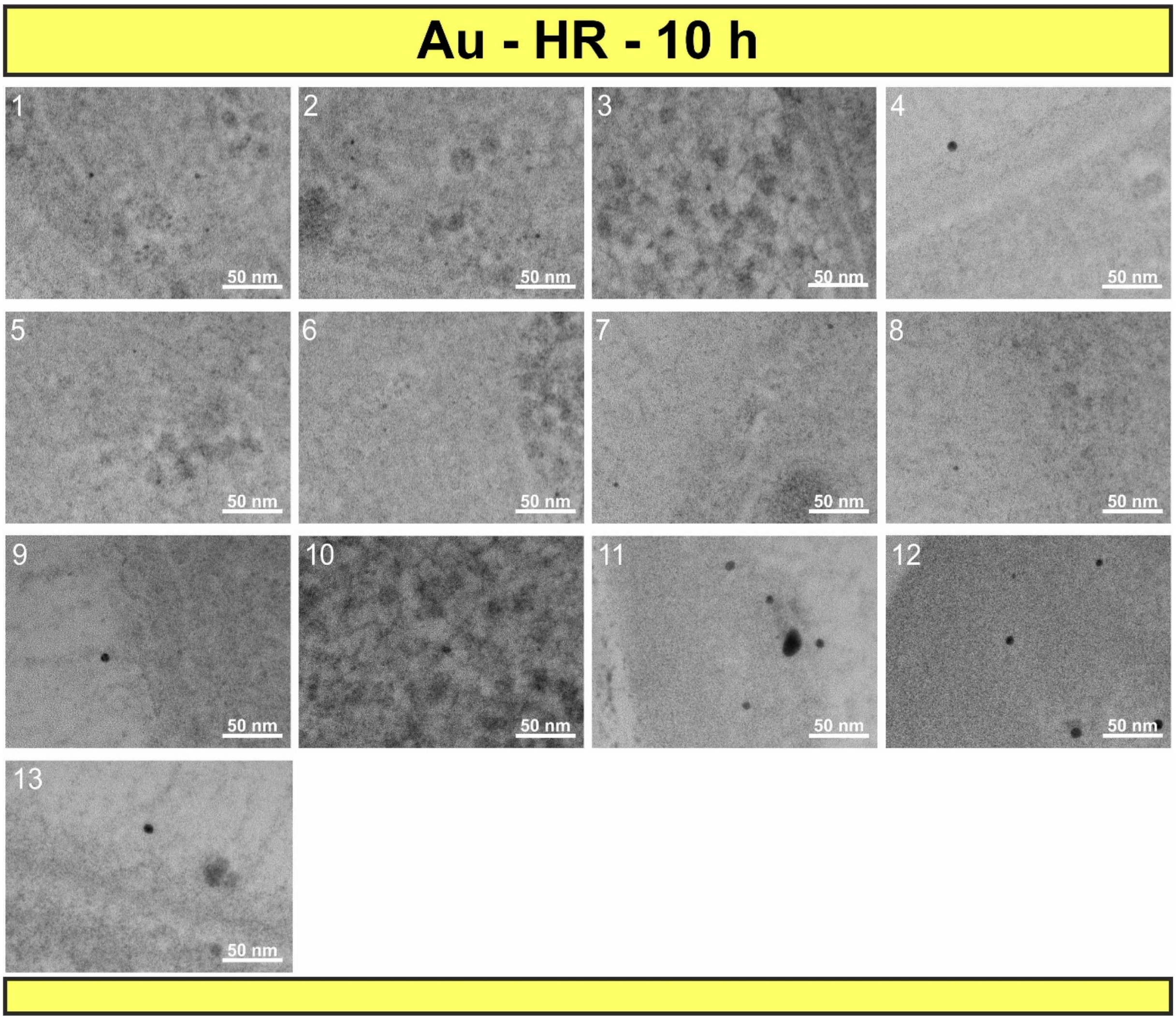

Figure S1 The PSDs of the AuNPs and their shape classification in Figure 4 for the high-resolution mode are based on the TEM images shown here.

Table S3 Settings of the respective TEM images showing AuNPs in the cells (showing for one magnification; here 500 nm bar, VC)

TEM image no.	Set scale factor [px/nm]	Particle counts	Size range [px]	Threshold [%]
14	1.838	622	15-500	0.73
15	1.372	213	15-200	0.78
16	1.838	537	10-150	0.46
17	1.836	535	15-250	1.50

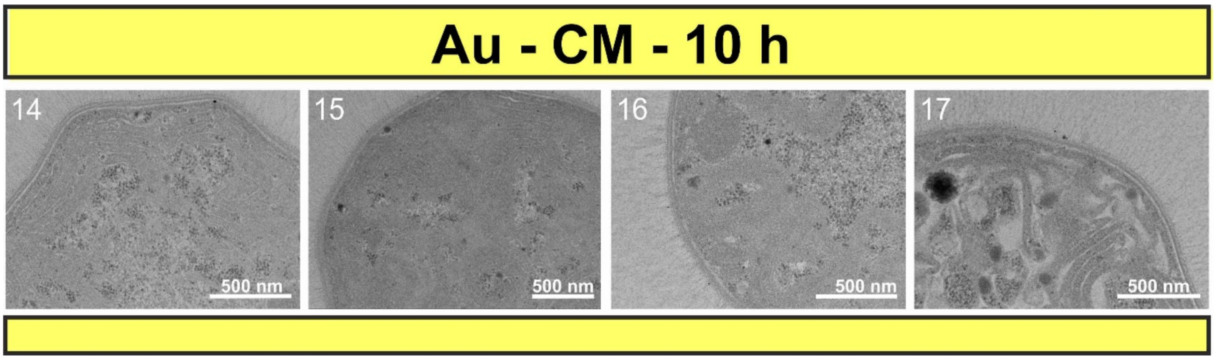

Figure S2 The PSDs of the AuNPs and their shape classification in Figure 4 for conventional mode are based on the TEM images shown here.

S.3 Biosynthesized Cerium nanoparticles (CeNPs)

Table S4 The TEM image settings used to determine the sizes and shape of CeNPs in Figure 6 are presented below. The symbol "∞" represents infinity.

TEM image no.	Set scale factor [px/nm]	Particle counts	Size range [px]	Threshold [%]
18	13.76	2	1800-∞	3.53
19	13.76	2	3500-∞	8.31
20	13.76	3	1000-∞	11.36
21	13.74	3	550-∞	17.60
22	9.16	3	1000-∞	16.80

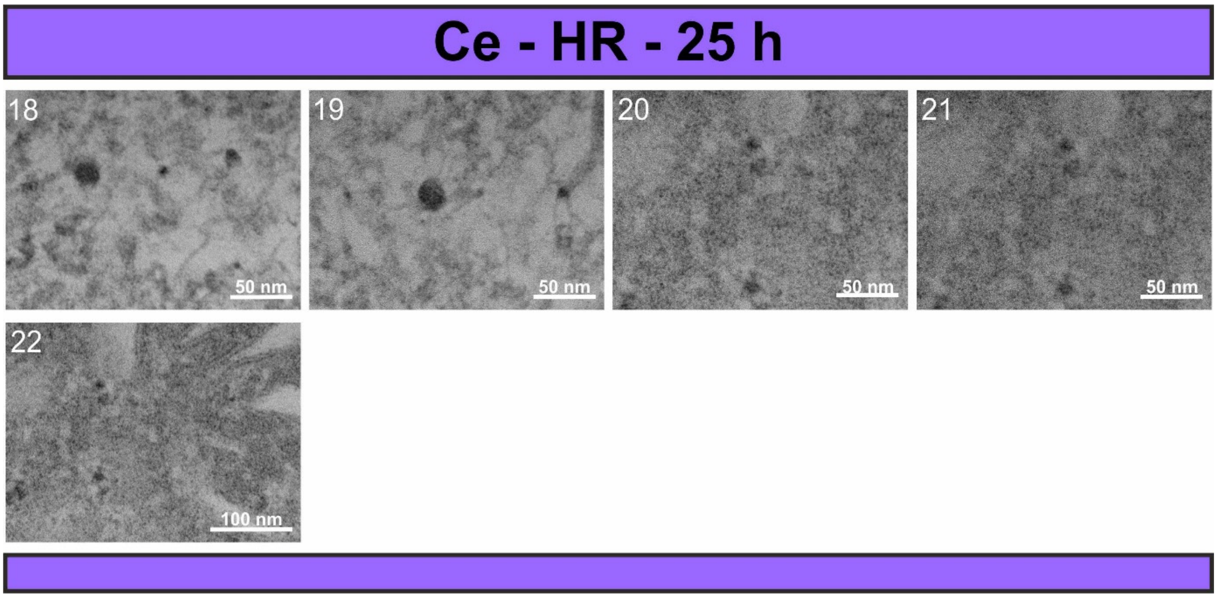

Figure S3 The PSDs of the CeNPs and their shape classification in Figure 6 for the high-resolution mode are based on the TEM images shown here.

Table S5 The TEM image settings used to determine the sizes of CeNPs by conventional mode.

TEM image no.	Set scale factor [px/nm]	Particle counts	Size range [px]	Threshold [%]
23	920	423	20-300	1.93
24	642	101	10-∞	0.31
25	918	262	10-300	0.25

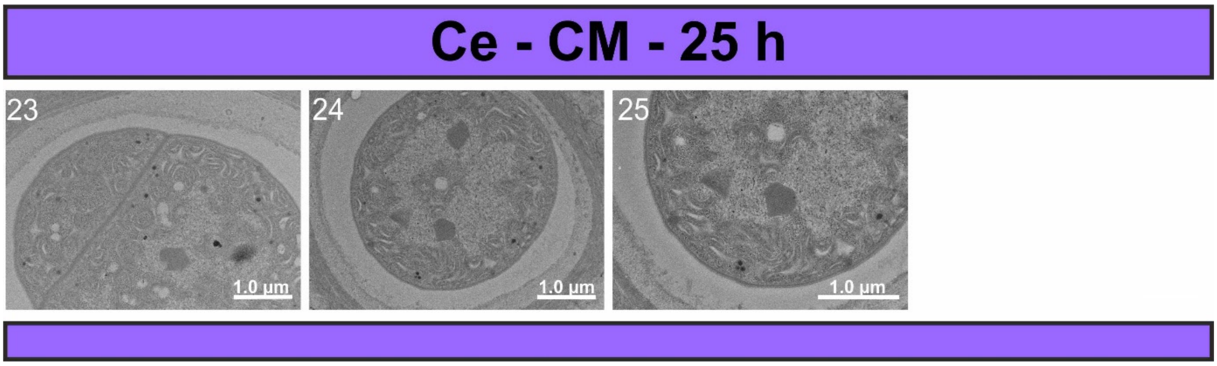

Figure S4 The PSDs of the CeNPs and their shape classification in Figure 6 for conventional mode are based on the TEM images shown here.
